# John Yudkin’s hypothesis: sugar is a major dietary culprit in the development of cardiovascular disease

**DOI:** 10.3389/fnut.2024.1407108

**Published:** 2024-07-04

**Authors:** Kenneth K.Y. Ting

**Affiliations:** ^1^Department of Immunology, University of Toronto, Toronto, ON, Canada; ^2^Toronto General Hospital Research Institute, University Health Network, Toronto, ON, Canada

**Keywords:** atherosclerosis, John Yudkin, inflammation, sucrose, sugar, uricemia, lipoprotein hyperproduction, fructose

## Abstract

To date, the risk of developing atherosclerosis has extended beyond Western countries and now affecting individuals from various ethnic backgrounds and age groups. Traditional risk factors of atherosclerosis, such as hypercholesterolemia, has been better controlled than before due to highly effective and inexpensive therapies at lowering plasma cholesterol levels. However, the role of reducing dietary cholesterol intake, as a public healthy strategy, in preventing the occurrence of cardiovascular mortalities has been recently challenged. Indeed, despite our continuous decline of dietary cholesterol intake within the last 50 years, the incidence of cardiovascular mortalities has continued to rise, thus raising the possibility that other dietary factors, such as fructose-containing sugars, are the major culprit. In the 1970s, John Yudkin first proposed that sugar was the predominant dietary factor that underlies the majority of cardiovascular mortalities, yet his hypothesis was dismissed. However, over the last 25 years substantial scientific evidence has been accumulated to support Yudkin’s hypothesis. The objectives of this review are to highlight Yudkin’s significant contribution to nutritional science by reviewing his hypothesis and summarizing the recent advances in our understanding of fructose metabolism. The metabolic consequences of fructose metabolism, such as fructose-induced uricemia, insulin resistance, lipoprotein hyperproduction and chronic inflammation, and how they are linked to atherosclerosis as risk factors will be discussed. Finally, the review will explore areas that warrant future research and raise important considerations that we need to evaluate when designing future studies.

## Introduction

Atherosclerosis remains one of the leading causes of worldwide cardiovascular mortalities. Within the past decades, new evidence in the field has spurred novel concepts that significantly alter our views on this illness, particularly our perspectives on the traditional risk factors of atherosclerosis (1). For instance, the risk factor profile has now shifted from the elevation of low-density lipoprotein (LDL) cholesterol levels to the elevation of triglyceride-rich lipoproteins (TGRL) and inflammatory pathways. This shift is attributed to the effective therapies on lowering plasma cholesterol levels, as well as the rise of favoring high-carbohydrate diet, which is associated with the rise of TGRL levels (1). Indeed, the intake of dietary fructose in US has drastically increased within the past 50 years, and its overconsumption has been proposed as a major culprit for the rise of many metabolic diseases, such as obesity, type 2 diabetes, atherosclerosis, as well as certain types of neurological dysfunction (2–6). The notion that sugar is the dietary factor that underlies various cardiovascular diseases was first proposed by John Yudkin in the 1970s (7), yet his hypothesis was dismissed. Fifty years later, there is a substantial body of scientific evidence to support his hypothesis (8). Our understanding of fructose metabolism has significantly advanced since then, although its mechanistic link to the development of atherosclerosis is only beginning to be appreciated (9, 10).

Initially thought to be only metabolized in the liver, recent advances in the metabolism field has now demonstrated that dietary fructose is metabolized first by intestine then followed by the liver (11, 12). Specifically, low concentration of fructose is metabolized by the intestine, while high concentration of fructose saturates the intestinal metabolic capacity, thereby allowing its leakage to the liver and colonic microbiota (11). The intrinsic metabolism of excessive fructose in the liver, but not the intestine, eventually leads to the development of many features of metabolic syndrome, such as hepatic steatosis (13) and hypertension (14), which are also risk factors for developing atherosclerosis. Therefore, in this review, I will review John Yudkin’s hypothesis, with the intent of highlighting his significant contribution to nutritional science and summarize the metabolic consequences of excess dietary fructose metabolism and how they are potentially linked to atherosclerosis as risk factors.

## John Yudkin’s hypothesis

In the 1960s, the death of the 34th American President Dwight D. Eisenhower from heart failure has sparked enormous research interest in determining the dietary factors that are responsible for inducing cardiovascular mortalities. Among all, two major school of thoughts were most popularized at that time, and they were proposed by Ancel Keys and John Yudkin, respectively. Keys on one hand hypothesized that total and saturated fat was responsible for the cause of cardiovascular diseases (15, 16), while Yudkin on the other hand proposed that dietary sugar was responsible (7, 17–21). Specifically, Yudkin first highlighted in his study in 1957 that the positive correlation between intake of sugar per year with coronary mortality was better than the consumption of total fat, including animal fat, butter fat, vegetable fat and margarine (20). Similar findings were also found in his subsequent study in the context of diabetic related mortalities (21). Notably, during this heated debate, the Sugar Research Foundation helped to downplay the evidence that showed sucrose consumption was a significant risk factor for developing heart disease (22). This eventually contributed to the public dismissal of Yudkin’s research and shaped the American Heart Association (AHA) dietary recommendation to reduce daily cholesterol consumption (19). For instance, it was recommended that Americans should lower the intake of saturated fats and replace it with mono- and polyunsaturated fats.

As a result of this dietary recommendation, we have observed a significant decline in dietary cholesterol intake in men and women, specifically from 500 mg/day for men and 320 mg/day for women in 1972 (23), to 348 mg/day for men and 242 mg/day for women in 2018 (24). Yet, the current incidence of cardiovascular diseases and obesity have continuously risen with no signs of stopping, which directly questions the role that dietary cholesterol plays in contributing to the current development of cardiovascular disease. In fact, this should not be surprising as the Seven Countries study, the infamous study that Keys formulated his hypothesis on, only demonstrated a correlation but not causation between intake of saturated fat and serum cholesterol levels (15). Indeed, meta-analysis of the effects of reducing saturated fat intake on improving cardiovascular health within the past decades have been conflicting and failed to reach a clear consensus (25–30).

Due to the lack of substantial evidence that demonstrate the beneficial effects of low-cholesterol diets on cardiovascular health, the AHA and the Dietary Guidelines Advisory Board in the United States eventually removed cholesterol as a nutrient of concern in Americans’ diet in 2015 (31). On the other hand, there is now strong scientific evidence suggesting that sugar (specifically fructose and fructose-containing sugars) is in fact the major culprit, thus supporting Yudkin’s hypothesis. This advocacy was first led by Dr. Robert Lustig (8, 32) and the number of studies that investigate how dietary fructose and fructose-containing sugars could worsen cardiovascular health have been continuously growing (9, 10, 33). For instance, recent epidemiological studies have now found that the consumption of total fructose from added sugar, but not from fruits and vegetables, were associated with a higher risk of coronary heart disease (CHD) (34). Similar results were also shown in individuals who frequently consumed sugar sweetened beverages, where they also had a greater risk of developing CHD when compared to infrequent consumers (35, 36). Overall, these studies demonstrate the important role that fructose overconsumption plays in driving the development of CHD, thus reflecting the urgent need to better understand its underlying mechanisms.

## The intestinal and hepatic metabolism of fructose

Human consumption of purified fructose alone is relatively rare as it is often consumed with glucose in the form of sucrose, which is a disaccharide composed of one glucose and one fructose molecule. Similarly, human consumption of fructose also takes place in the form of high-fructose corn syrup (HFCS), which is composed of either 42% or 55% of fructose, with the rest being glucose. Nonetheless, upon the ingestion of sucrose or HFCS, the brush border of the small intestine secretes sucrase-isomaltase to cleave them into free glucose and fructose molecules (37, 38). Interestingly, sucrase-isomaltase can also utilize a distinct cleavage site to cleave maltose, a disaccharide link by two glucose molecules, into two free glucose molecules (37, 39).

**Figure 1:**
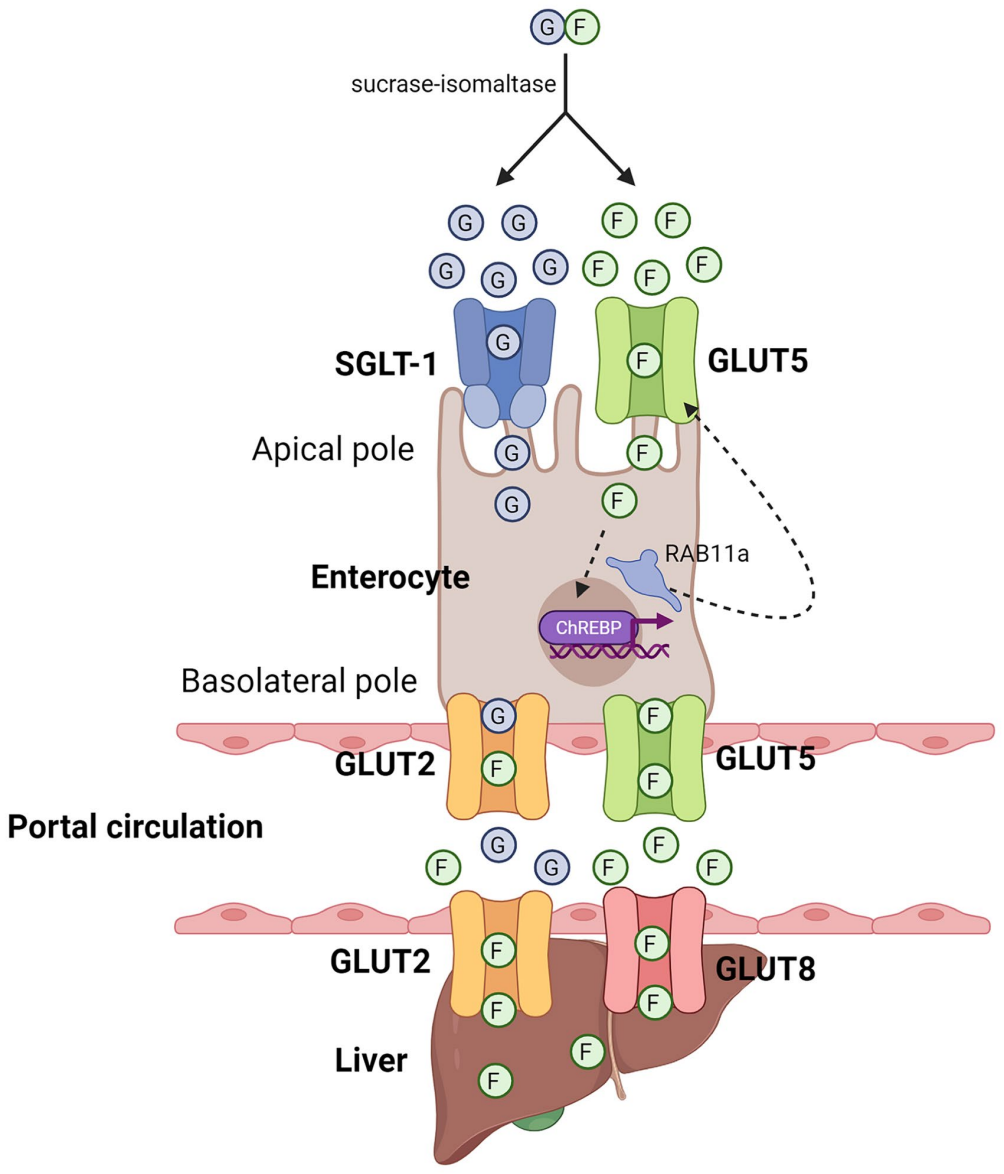
Entry of fructose in enterocytes and hepatocytes. Diagram that illustrates the respective entry of glucose and fructose in enterocytes and hepatocytes. Sucrase-isomaltase breaks down sucrose into free glucose and fructose molecules. Glucose and fructose enter the enterocytes through the apical pole through SGLT-1 and GLUT5, respectively. At the basolateral pole, fructose can leave enterocytes into the portal circulation through GLUT2 and GLUT5, which can then enter hepatocytes through GLUT2 and GLUT8. Green circle denotes free fructose molecules, blue circle denotes free glucose molecules. Created with Biorender.com.

Upon the cleavage of sucrose or HFCS into free monosaccharides, fructose and glucose enter the apical side of enterocytes through SLC2A5 (GLUT5), a passive transporter, and sodium-glucose co-transporter 1 (SGLT-1), an active co-transporter, respectively (40–42) (Figure 1). After entry, the metabolism of fructose increases the expression of GLUT5 to enhance the further uptake of fructose. Specifically, past studies have found that fructose metabolism in enterocytes activated carbohydrate-responsive element-binding protein (ChREBP)-dependent transcription of GLUT5 (41, 43), followed by increased endosomal tracking of GLUT5 to the apical membrane in a Ras-related protein-in-brain 11a (RAB11a)-dependent manner (41), which eventually increased the surface expression of GLUT5. Apart from this, fructose absorbance can also be enhanced by the uptake of other molecules, such as glucose, alanine, proline, and glutamine, with glucose being the most potent one (44, 45). Mechanistically, it has been proposed that thioredoxin-interacting protein (TXNIP), which is known to regulate glucose homeostasis, interacts with GLUT5 and promotes fructose uptake, thereby linking glucose and fructose metabolism together in enterocyte (46). However, the precise molecular events that illustrates how TXNIP enhance fructose uptake via interacting with GLUT5 remains to be determined.

**Figure 2:**
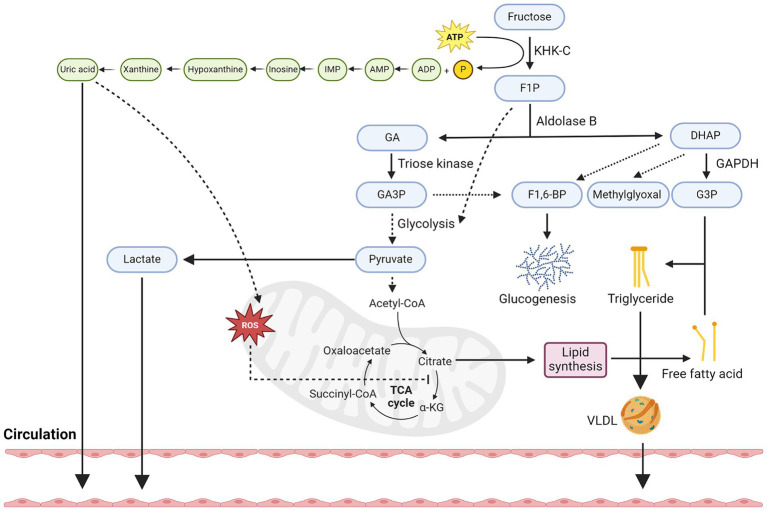
Hepatic metabolism of fructose. Diagram that illustrates key metabolic steps of fructose metabolism in hepatocytes. Firstly, fructose is constitutively phosphorylated by KHK-C into F1P and leads to the depletion of ATP pools. This in turn increases pools of ADP and eventually the production and secretion of uric acid into the circulation. Uric acid can also stimulate oxidative stress in mitochondria and block aconitase activity, leading to citrate accumulation. Secondly, F1P can be converted to GA and DHAP by aldolase, in which GA can be phosphorylated by triose kinase into GA3P. GA3P can participate in glycolysis and converted to pyruvate or combine with DHAP to form F1-6-BP and enter the glucogenesis pathway. Thirdly, fructose-derived pyruvate can be transformed into lactate and secreted into circulation or participate in the TCA cycle and further increases the levels of citrate. Citrate can then be converted back to acetyl-CoA and participate in the generation of fatty acids, although the acetate produced from fructose metabolism in gut microbes can also contribute to the lipogenic pools of acetyl-CoA. Fourthly, F1P-derived DHAP can also be converted to G3P, which when combined with free fatty acids, can then be used to synthesize triglyceride. The synthesis of triglycerides can be stored as lipid droplets or secreted into the circulation as VLDL. Created with Biorender.com.

If the amount of fructose and glucose taken up by enterocytes exceed their maximal capacity of metabolism, the excess fructose and glucose molecules will exit through the basolateral side of enterocytes into the portal circulation via GLUT5 and GLUT2, respectively, (40, 47, 48), although it has also been shown that fructose can also exit through GLUT2 (49). Through the portal circulation, fructose can subsequently enter hepatocytes through GLUT2 (50–52), as well as GLUT8 (53), but not through GLUT5 as it is not well expressed in hepatocytes (52). Similar to its metabolism in enterocytes, fructose in hepatocytes is first phosphorylated by ketohexokinase (KHK), specifically by KHK-C, one of the two alternatively spliced isoforms of KHK that is expressed in liver, small intestine, and kidney (54) (Figure 2). Unlike its other isoform, KHK-A, which differs from KHK-C by one exon, KHK-C has a higher affinity for fructose than KHK-A (31, 55). Therefore, upon its phosphorylation by KHK-C, fructose is rapidly converted into fructose-1-phosphate (F1P). Since the activity of KHK is not subjected to feedback inhibition, its phosphorylation of fructose to F1P is unrestricted, and leads to a constitutively decline of ATP pools and rise of ADP and inosine monophosphate (IMP) pools, which ultimately leads to the formation of uric acid and mitochondrial reactive oxygen species (mtROS) (12, 56).

After F1P is generated, it is converted to glyceraldehyde (GA) and dihydroxyacetone phosphate (DHAP) by aldolase B. GA is then phosphorylated by triose kinase into glyceraldehyde 3-phosphate (GA3P), which is subsequently converted into phosphoenolpyruvate and pyruvate through a series of enzymatic reactions. The production of pyruvate can be converted lactate via lactate dehydrogenase or enter the tricarboxylic acid (TCA) cycle via pyruvate dehydrogenase. Apart from the production of pyruvate, GA can also combine with F1P-derived DHAP to form fructose-1-6-bisphosphate (F1,6-BP) and enter the gluconeogenic pathway in a ChREBP-dependent manner (57, 58). Finally, F1P-derived DHAP can also be converted to glycerol-3-phosphate (G3P) by Glyceraldehyde 3-phosphate dehydrogenase (GAPDH), as well as methylglyoxal by methylglyoxal synthase. Methylglyoxal has been shown to further inhibit acetyl-CoA from entering the mitochondria and inhibits AMP-activated protein kinase (AMPK) signaling, thus promoting fatty acid synthesis over oxidation.

Once pyruvate enters the TCA cycle, it specifically raises the levels of citrate due to mtROS-mediated inhibition of aconitase (56). This increased level of citrate is then converted to acetyl-CoA by ATP Citrate Lyase (ACLY), and eventually malonyl-CoA by Acetyl-CoA carboxylase 1 (ACC1). Malonyl-CoA can be used to synthesize C16 and C18 fatty acids, such as palmitate, by fatty acid synthase (FASN). These fatty acids can be secreted into the circulation, or when conjugated with DHAP-derived G3P, lead to the formation of triglyceride (TG) and secreted into circulation as very low-density lipoproteins (VLDL) or stored as lipid droplets. Apart from this, it is also important to highlight that recent research has shown that acetate secreted by gut microbes post excess fructose metabolism can also contribute to the lipogenic pools of acetyl-CoA in hepatocytes (59). This process is independent of hepatocyte ACLY activities and suggests that intrinsic fructose metabolism of gut microbes also participates in hepatic lipogenesis.

Finally, the intrinsic metabolism of fructose in hepatocytes also enhances its rate of glycolysis, specifically, through the production of F1P. Although the rate of glycolysis is tightly regulated based on the cellular energy state (ATP levels) at the level of phosphofructokinase (PFK), KHK-C-mediated depletion of ATP levels, as well as uric acid (60), can trigger the stimulation of PFK activity and thus promotes a high glycolytic flux in hepatocytes. Notably, F1P itself can also allosterically activate glucokinase-regulatory protein and liver-type pyruvate kinase, which both further enhance the uptake of glucose and its metabolism. Taken together, fructose metabolism in hepatocytes can contribute to multiple cellular energetic processes, including increased *de novo* lipogenesis, glycolysis, and gluconeogenesis. Metabolite tracing experiments with isotopes have supported this and revealed that upon fructose oxidation, 25% is converted to lactate, 15% is converted to glycogen, 50% is converted to glucose, with the rest being converted to triglyceride (61–64).

## Fructose-induced uricemia

As previously described, upon excess dietary fructose-containing sugar consumption, the uncontrolled phosphorylation of fructose by KHK-C in hepatocytes will lead to its depletion of ATP and in turn increase the formation of uric acid. The deposition of uric acid from hepatocytes into the circulation can rapidly elevate its levels up to 4 mg/dL (65, 66), and extend into the postprandial phase (67). Interestingly, fructose is the only monosaccharide that induces uricemia and its deleterious effect has been previously demonstrated. For instance, Nakagawa et al. have first demonstrated that fructose-induced uricemia underlie features of metabolic syndrome, such as hypertension, and that lowering the concentration of uric acid by using uricosuric agent or xanthine oxidase inhibitor could confer protective effects (14).

Hypertension and uricemia are both key risk factors for atherosclerosis (68–70), and fructose-induced uricemia has been long-associated with hypertension (71, 72), most likely through inducing endothelial dysfunction. It is well established that nitric oxide (NO) produced by eNOS in endothelial cells is critical for maintaining blood pressure by inducing smooth muscle cells dilation. However, in the context of hyperuricemia, uric acid can inhibit eNOS-derived NO production levels in both *in vitro* and *in vivo* models (73, 74), thereby contributing to hypertension. Mechanistically, uric acid activates NADPH-mediated oxidative stress in endothelial cells, and that the production of reactive oxygen species (ROS) impairs the function of eNOS and thus its production of NO (75, 76). Apart from inducing oxidative stress, uric acid can also inhibit eNOS function by inducing the expression of C-reactive proteins (CRP). Specifically, a study performed by Kang et al. have shown that uric acid-induced CRP in endothelial cells could inhibit the release of NO, and that NO levels could be in turn normalized with the treatment of anti-CRP antibodies (73). Finally, in addition to endothelial dysfunction, fructose metabolism has also been linked to hypertension through other mechanisms, such as sympathetic hyperactivity (77), inhibition of the effect of acetylcholine and prostacyclin (78, 79), and the enhancement of the effect of vasoconstrictor substances (80).

Apart from hypertension, fructose-induced uricemia can be linked to atherosclerosis through chronic inflammation and the enhanced production of chemokines that attract the recruitment of monocytes. Indeed, past studies have shown that lowering uric acid concentration has significantly reduced macrophage infiltration and TNFα expression (81). Mechanistically, uric acid has been shown to induce the formation of NLRP3 inflammasome (82), as well as activating p38 MAPK signaling pathways. It is well established that NLRP3 inflammasome is critical for the production of IL-1β, which has been recently shown to be involved in the development of atherosclerosis (83). On the other hand, the activation of p38 MAPK signaling is known to be pro-atherogenic due to its downstream cascades involving the production of adhesion molecules and induction of angiogenesis (84). Taken together, fructose-induced uricemia can be linked to the progression of atherosclerosis through endothelial dysfunction, enhanced inflammation, and increased recruitment of monocytes into the intima.

## Fructose-induced insulin resistance

Long-term excess hepatic fructose metabolism can also induce insulin resistance (85–87), which is another strong predictor of atherosclerosis (88–90), specifically inducing hepatic insulin resistance prior to whole body insulin resistance (91, 92). In recent years, several mechanisms have been proposed to link fructose metabolism with hepatic insulin resistance, in which most of these pathways are associated with the consequential effects of *de novo* lipogenesis post hepatic fructolysis. As previously described, hepatic fructose metabolism is intrinsically lipogenic (93), and that various lipotoxic intermediates and fatty acids are created during this process. Notably, several of these intermediates, such as diacylglycerol (DAG) and ceramides, have been shown to promote hepatic insulin resistance (94–96).

DAG is the immediate precursor of TG as Diacylglycerol O-acyltransferase catalyzes the conversion of DAG with fatty acyl CoA into TG. In the context of excess hepatic lipid synthesis, DAG was shown to activate protein kinase C (PKC)ε and inhibit hepatic insulin signaling. Specifically, DAG induces PKCε translocation to the plasma membrane and phosphorylates insulin receptor tyrosine kinase (IRTK) at Thr1160, thus inhibiting its activity and insulin-mediated phosphorylation on insulin receptor substrate 2 (IRS2) (97, 98). More importantly, knocking down PKCε with an antisense oligonucleotide has reversed the effects of fat-induced hepatic insulin resistance in rats (98), which directly supports the DAG-PKCε induced hypothesis of hepatic insulin resistance.

Apart from DAG, ceramides have also been proposed to promote hepatic insulin resistance as well. Ceramides are sphingolipids that are produced from sphingosine and fatty acids, and generally play an important role in regulating cell membrane stabilization and distribution of signaling proteins (96). In the context of hepatic insulin resistance, it was shown that ceramide can either activate PKCζ and block protein kinase B (AKT) from participating insulin-mediated signaling, or activate protein phosphatase 2A, which is responsible for the dephosphorylation and thus inactivation of AKT (99, 100). In addition to lipotoxic intermediates, other metabolites secreted during hepatic fructolysis, such as fructose-derived lactate, are also linked to insulin resistance. As previously described, lactate accounts for 25% of secreted metabolites post fructolysis, and elevation of plasma lactate levels is known to induce peripheral insulin resistance (101). Mechanistically, lactate infusion has suppressed the ability of insulin to stimulate IRS2-associated phosphatidylinositol 3-kinase and AKT activities in skeletal muscle cells (102). Taken together, these studies have collectively demonstrated that metabolites post fructolysis in the liver are linked to both hepatic and peripheral insulin resistance.

## Fructose-induced lipoprotein hyperproduction

A significant connection between excess fructose consumption and atherosclerosis is fructose-induced lipoprotein hyperproduction in the liver, such as very low-density lipoproteins (VLDL). VLDL, a long-established marker for atherosclerosis (103, 104), are mainly produced in the liver and their synthesis is dependent on the availability of lipid substrates, such as triglycerides, fatty acids and Apolipoprotein B100 (ApoB) (105–108). As previously described, excess hepatic metabolism of fructose significantly contributes to *de novo* lipid synthesis, hence leading to the overproduction of VLDL into the circulation, eventually raising plasma triglyceride levels. Indeed, a past study has shown that the levels of plasma ApoB, small dense LDL and oxidized LDL were increased in subjects that that have consumed fructose (93), thus strengthening the association between postprandial hypertriglyceridemia and proatherogenic conditions.

The elevation of lipid biosynthesis is a consequence of activating the expression of genes related to lipid synthesis, as well as suppressing the expression of genes related to fatty-acid oxidation. For instance, sterol regulatory element binding protein (SREBP), the master transcription factor that regulates the synthesis of cholesterol and fatty acid, one of its isoforms (SREBP-1) was found to be activated in mice post 60% fructose diet (109). Mechanistically, the binding activity of SREBP-1 was found to be regulated by insulin signaling, specifically through the MAPK signaling (110), although some studies have also shown SREBP-1 can be activated independently of insulin (111, 112). Apart from SREBP-1, ChREBP is another well-known transcription factor that regulates the expression of lipogenic genes upon activation by carbohydrate metabolites in the liver. A past study has shown that high-fructose diet in mice activated hepatic ChREBP and expression of its targeted genes involved in lipid synthesis, a process also independent of insulin signaling (58). Finally, the inhibited expression of genes involved in fatty acid oxidation also contributes to the overproduction of lipoproteins. For example, the α member of the Peroxisome proliferator-activated receptors (PPAR) family regulates fatty acid oxidation genes, and was shown to be suppressed in fructose-fed rats (113). In addition to transcriptional regulation, past studies have also shown that fructolysis-derived methylglyoxal, as well as uric acid inhibiting adiponectin secretion from adipocytes, contributed to the inhibition of AMPK signaling, which further inhibits fatty acid oxidation in hepatocytes. Taken together, these studies have demonstrated that both transcriptional and post-transcriptional mechanisms are involved in regulating the enhancement of hepatic lipid synthesis post excess fructose feeding.

## Fructose-induced chronic inflammation

The success of the CANTOS trial, which targets interlukin-1β with a therapeutic monoclonal antibody, supports the notion that reducing inflammation independent of lowering lipid levels can lower the risk of cardiovascular diseases. The result of the trial also implicates that targeting other inflammatory pathways, such as the ones mediated by interlukin-6 or tumor necrosis factor alpha (TNFα) (83), can display therapeutic benefits, yet the molecular mechanisms behind how inflammation is initiated in atherosclerosis remains controversial (1). For instance, past studies have shown that the accumulation of oxLDL or cholesterol crystals in macrophages is an intrinsically inflammatory process (114, 115). However, other studies have shown that oxLDL or cholesterol accumulation in macrophages significantly suppressed their inflammatory responses due to rewiring of their metabolism (116–119). More recently, transcriptomic analyses of mouse and human atherosclerotic lesions have revealed that foamy macrophages are in fact less inflammatory than non-foamy macrophages (120, 121). Taken together, these studies support the notion that while hypercholesterolemia is a key risk factor of atherosclerosis, oxLDL or cholesterol do not intrinsically activate inflammation in Mφs both *in vitro* and *in vivo*.

On the contrary, there is an increasing appreciation in the immunometabolism field that fructose metabolism and macrophage inflammatory responses are connected. For instance, it has been shown that intrinsic fructose metabolism of macrophages can promote their inflammatory responses in a glutamine-dependent oxidative metabolism manner (122). In addition to its intrinsic inflammatory effects, excess fructose metabolism has been recently demonstrated to cause endotoxemia and promote systemic inflammation (13). Mechanistically, this study has shown that excess fructose metabolism could trigger intestinal barrier deterioration and promote the leakage of microbial-derived products into the portal circulation. These products could subsequently activate residential macrophages in the liver (Kupffer cells), stimulate their production of TNF-α in a Toll-like receptor 4-dependent manner, in which the secreted TNF-α could then stimulate *de novo* lipogenesis in hepatocytes (13). Similar findings were also reported by Kavanagh et al. where the authors found that a high fructose diet induced gut microbial translocation, endotoxemia and liver damage in non-human primates (123). Collectively, these studies have demonstrated that high fructose diets can intrinsically and extrinsically promote macrophage inflammatory responses through metabolic means.

Apart from macrophages, fructose has also been linked to inflammation through the formation of advanced glycation end (AGEs) products and its subsequent activation of its receptor, known as receptor for advanced glycation end product (RAGE). Interestingly, this effect has been shown in multiple cell types, such as dendritic cells (124) and endothelial cells (125), which implies that the underlying molecular mechanism is conserved, and its downstream inflammatory effect is likely systemic. In general, the formation of AGE by fructose involves the interaction between the reactive carbonyls in fructose and the amino groups of proteins, DNA and lipids, known as the Millard reaction. Unlike glucose, fructose is significantly more reactive in Maillard reaction due to its keto group and its enhanced stability in its open chain formation (126–130). Post Maillard reaction, fructose generates early glycation products which undergo a series of further conversion into AGEs (131). In hepatocytes, where fructose metabolism largely takes place, the formation of AGEs has been detected by Liquid chromatography–mass spectrometry (LC–MS) in fructose-drinking mice, and the by-products of AGEs formation, such as N-carboxymethyllysine, are linked to the enhancement of *de novo* lipid synthesis in hepatocytes (132).

Apart from enhancing lipid synthesis, the classical consequence of AGEs is the binding of RAGE and the initiation of downstream inflammatory signaling cascades. For instance, exposing dendritic cells with fructose (15 mM) has led to the increased formation of AGEs, and its binding to RAGE caused an increased production of inflammatory cytokines, such as IL-1β and IL-6, in a NF-κB-dependent manner (124). This increased inflammatory response is accompanied by a shift of metabolism from oxidative metabolism to glycolysis. Similarly, exposing endothelial cells with fructose has induced AGEs formation, and subsequently led to the formation of oxidative stress and inflammatory reactions also in a NF-κB-dependent manner (133, 134). Overall, these studies collectively support the notion that fructose-induced AGEs formation is likely a systemic inflammatory process that occurs in both immune and non-immune cell types.

## Conclusion and future perspectives

**Figure 3:**
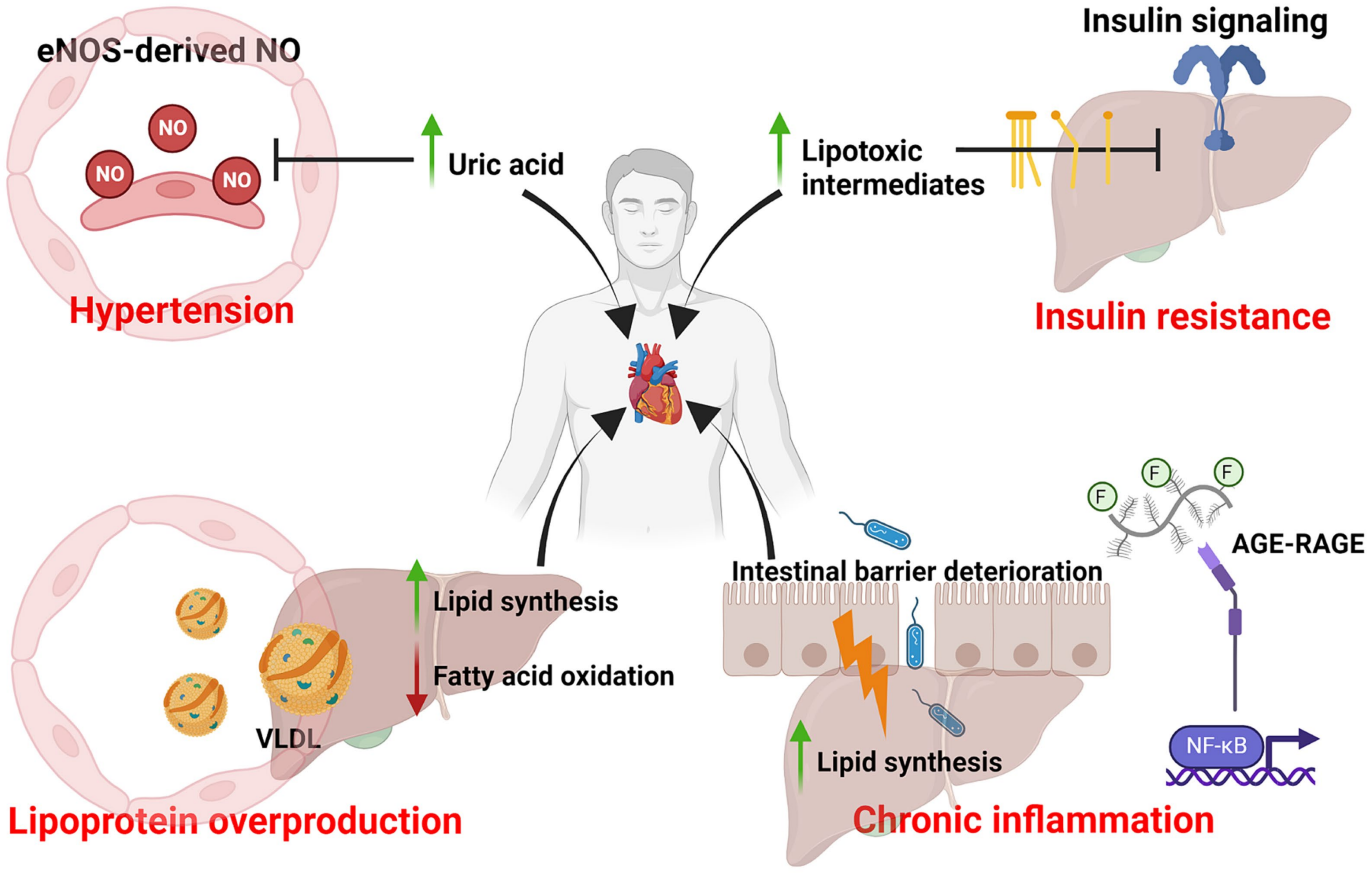
Excess dietary fructose metabolism and risk factors of atherosclerosis. Diagram that summarizes the various metabolic consequences of excess dietary fructose metabolism and its association with atherosclerosis as risk factors. This includes uricemia-induced hypertension, lipotoxic intermediates-mediated insulin resistance, lipoprotein overproduction and chronic inflammation. Created with Biorender.com.

Within the past decades, our consumption of dietary fat has significantly declined (23, 24) yet the incidence of cardiovascular diseases has continued to rise, thus implicating the possibility that dietary fat may not be the culprit behind the prevailing cardiovascular mortalities. On the other hand, dietary fructose consumption has tremendously increased and positively correlated with the rise of metabolic diseases, including but not limited to diabetes, obesity and atherosclerosis (5, 6, 8). In the 1970s, John Yudkin first proposed the notion that sugar is the dietary factor that contributes to cardiovascular illnesses (7, 17, 20). Although his hypothesis was initially dismissed, researchers have been revisiting his hypothesis for the last 25 years and have accumulated strong and convincing evidence to support it. To date, our understanding of fructose metabolism, specifically in the intestine and liver, has significantly advanced. However, the mechanistic link between excess fructose metabolism and the development of cardiovascular diseases, such as atherosclerosis, is only beginning to be appreciated. Furthermore, how excess fructose metabolism contributes to the induction of inflammation in atherosclerosis remains to be determined. In this review, I have provided an overview of the metabolic consequences after excess fructose metabolism and how these consequences are potentially linked to atherosclerosis as risk factors (Figure 3).

Upon reviewing the current literature on fructose-related studies, several important considerations need to be taken during the design of mechanistical studies to ensure the results are not confounded by third-variables, one of which is the administration of fructose. In general, for *in vivo* studies, many investigators typically feed rodents with fructose-rich drinks or diet *ad libitum*. However, this may pose a problem as fructose is intrinsically addictive (135) and fructose-fed rodents may consume more calories than the control diets. Therefore, isocaloric diets, such as administered through oral gavage, is always a better approach as it prevents excess caloric intake from confounding the experimental results. Apart from this, the type of fructose diet, such as purified fructose or in the form of sucrose, should also be considered. Since humans do not typically consume fructose in its purified form, and past studies have already shown that the uptake of fructose is enhanced in the presence of glucose (44), this suggests that a sucrose diet should be favored over a purified fructose diet to ensure physiological relevance. Since the sucrase-isomaltase expressed in the intestine can also cleave maltose, in addition to sucrose, a maltose diet can be used as a control diet when compared against the experimental sucrose diet. Interestingly, a recent study has also shown that sucrose-fed mice with drinking water versus solid diet yielded differential metabolic results despite consuming equivalent amounts of sucrose (136). In humans, it has been suggested that liquid sugars are more detrimental with regards to body weight than its solid forms, most likely due to the fact that sugar beverages produce less satiety and thus leads to decreased dietary energy compensation and increased intake (137–139). However, only one clinical dietary intervention study has been conducted comparing the effects of sustained consumption of liquid versus solid sugar, and a significant group difference in body weight was not observed (138). Importantly, a recent prospective study found that both added sugar in food and added sugar in beverage increased the risk of developing metabolic syndrome during a 30-year follow-up (140). Therefore, more studies comparing liquid versus solid sugar are needed to determine whether the consumption of liquid sugar is more detrimental than solid sugar and investigators need to be aware of this. Finally, investigators should also consider the type of cells they are investigating in relation to fructose metabolism. In this review, some *in vitro* studies have incubated their cells of interest with fructose at a concentration that is unlikely to be physiologically relevant. On the other hand, investigators should also be aware that certain organs, despite not being directly related to fructose metabolism, do endogenously synthesize fructose post hyperglycemic conditions, such as the brain (141). This suggests that the effects of dietary fructose consumption can extend to other cell types beyond their metabolizing organs.

In 1972, Yudkin wrote in his book, *Pure, White, and Deadly: How Sugar Is Killing Us and What We Can Do to Stop It,* “if only a small fraction of what is already known about the effects of sugar were to be revealed in relation to any other material used as a food additive, that material would promptly be banned” (18). While this message was ignored for 25 years, the book was republished in 2012 with a forward by Dr. Robert Lustig. Although our current understanding of how excess fructose metabolism is causally linked to cardiovascular mortality is still rudimentary, its detrimental metabolic consequences have already been revealed and continuously supported by ongoing research. Continued research is warranted to further elucidate the mechanistic link between fructose metabolism and cardiovascular disease.

## Author contributions

KT: Writing – original draft, Writing – review & editing.
